# Supplementary material for: AB-free Kava reduces anxiety-like behavior without preventing nicotine-induced exploration suppression in mice
Source: Front Pharmacol. 2026 Oct 14;17:1981677. doi: 10.3389/fphar.2026.1981677 (PMC13713732; doi:10.3389/fphar.2026.1981677)
Supplement: Supplementary file 1 [file Supplementaryfile1.docx]

Supplemental Figures

A

B

**Figure S1. Apparatus used for open field testing.** (A) AccuScan open field apparatus. Clear Plexiglas activity cages (40 × 40 × 30 cm; L × W × H) equipped with infrared beam panels for automated detection of locomotor activity and time in center (VersaMax™ software). (B) Circular open field arena (55 cm diameter, 42 cm height) with a white floor and dark blue walls. Four arenas shown simultaneously tracked using Noldus EthoVision XT software.

**Figure S2. Absolute body weight over time by sex, diet, and treatment during the study.** Body weight (g) was measured across 24 timepoints from Day 1 to Day 57 in male and female mice fed either a control or kava-containing diet and treated with either saline or nicotine. The arrow indicates the start of the dietary intervention (Day 15); body weights prior to this point represent the pre-diet baseline period. Data are presented as mean ± SEM. N = 8–9 per group.

**Figure S3. Body weight expressed as a percentage of pre-diet baseline.** Body weight at each post-diet timepoint (Day 18–Day 57) was normalized to the body weight of each animal (Day 14 = 100%) to account for individual differences in starting weight. Data are shown for male and female mice fed either a control or kava-containing diet and treated with either saline or nicotine. The statistical analysis did not reveal a significant effect of diet or nicotine treatment on normalized body weight, while a significant effect of sex was observed, with males showing greater relative weight gain than females. Data are presented as mean ± SEM. N = 8–9 per group.

Supplemental Tables

**Supplementary Table S1. Sensitivity analysis accounting for cage as a random effect.**

As a secondary analysis, each primary outcome was additionally analyzed with a linear mixed-effects model (MIXED procedure, restricted maximum likelihood) with diet, treatment, sex, and their interactions as fixed effects and cage as a random intercept. Denominator degrees of freedom were estimated by the Satterthwaite method. The ANOVA columns reproduce the three-way ANOVA reported in the main text, in which the individual mouse was the unit of analysis; values for the treatment block are the between-subjects effects of the repeated-measures ANOVA (mean of five sessions). Cage ICC is the intraclass correlation coefficient, the proportion of residual variance attributable to cage. Effects that were significant in the ANOVA but not in the cage-adjusted model are shown in bold.

|  | | **ANOVA (main text)** | | | **Cage-adjusted mixed model** | | |  |
| --- | --- | --- | --- | --- | --- | --- | --- | --- |
| **Outcome** | **Effect** | **F** | **df** | **p** | **F** | **df** | **p** | **Cage ICC** |
| Distance, treatment block (n = 65) | Diet | 6.719 | 1, 57 | **0.012** | 4.527 | 1, 9.0 | **0.062** | 0.13 |
|  | Treatment | 10.077 | 1, 57 | 0.002 | 6.907 | 1, 11.3 | 0.023 |  |
|  | Sex | 0.464 | 1, 57 | 0.498 | 0.356 | 1, 9.0 | 0.566 |  |
|  | Diet × treatment | 1.071 | 1, 57 | 0.305 | 0.931 | 1, 11.3 | 0.355 |  |
|  | Diet × sex | 0.077 | 1, 57 | 0.782 | 0.041 | 1, 9.0 | 0.844 |  |
|  | Treatment × sex | 2.057 | 1, 57 | 0.157 | 1.706 | 1, 11.3 | 0.217 |  |
|  | Diet × treatment × sex | 0.270 | 1, 57 | 0.605 | 0.131 | 1, 11.3 | 0.724 |  |
| Center time, treatment block (n = 65) | Diet | 4.419 | 1, 57 | **0.040** | 3.959 | 1, 8.9 | **0.078** | 0.04 |
|  | Treatment | 11.680 | 1, 57 | 0.001 | 10.348 | 1, 11.3 | 0.008 |  |
|  | Sex | 2.280 | 1, 57 | 0.137 | 1.987 | 1, 8.9 | 0.192 |  |
|  | Diet × treatment | 6.043 | 1, 57 | 0.017 | 5.566 | 1, 11.3 | 0.037 |  |
|  | Diet × sex | 1.132 | 1, 57 | 0.292 | 1.026 | 1, 8.9 | 0.338 |  |
|  | Treatment × sex | 0.064 | 1, 57 | 0.802 | 0.071 | 1, 11.3 | 0.795 |  |
|  | Diet × treatment × sex | 0.510 | 1, 57 | 0.478 | 0.496 | 1, 11.3 | 0.495 |  |
| Distance, challenge 1 (n = 65) † | Diet | 1.554 | 1, 57 | 0.218 | 1.554 | 1, 57 | 0.218 | 0.00 |
|  | Treatment | 8.666 | 1, 57 | 0.005 | 8.666 | 1, 57 | 0.005 |  |
|  | Sex | 0.742 | 1, 57 | 0.393 | 0.742 | 1, 57 | 0.393 |  |
|  | Diet × treatment | 1.805 | 1, 57 | 0.184 | 1.805 | 1, 57 | 0.184 |  |
|  | Diet × sex | 2.814 | 1, 57 | 0.099 | 2.814 | 1, 57 | 0.099 |  |
|  | Treatment × sex | 2.659 | 1, 57 | 0.108 | 2.659 | 1, 57 | 0.108 |  |
|  | Diet × treatment × sex | 4.790 | 1, 57 | 0.033 | 4.790 | 1, 57 | 0.033 |  |
| Center time, challenge 1 (n = 65) | Diet | 1.114 | 1, 57 | 0.296 | 0.546 | 1, 9.4 | 0.478 | 0.37 |
|  | Treatment | 17.019 | 1, 57 | < 0.001 | 9.169 | 1, 11.2 | 0.011 |  |
|  | Sex | 2.436 | 1, 57 | 0.124 | 0.882 | 1, 9.4 | 0.371 |  |
|  | Diet × treatment | 5.498 | 1, 57 | **0.023** | 1.929 | 1, 11.2 | **0.192** |  |
|  | Diet × sex | 6.966 | 1, 57 | **0.011** | 2.660 | 1, 9.4 | **0.136** |  |
|  | Treatment × sex | 0.668 | 1, 57 | 0.417 | 0.120 | 1, 11.2 | 0.736 |  |
|  | Diet × treatment × sex | 4.048 | 1, 57 | **0.049** | 2.513 | 1, 11.2 | **0.141** |  |
| Distance, challenge 2 (n = 65) | Diet | 0.000 | 1, 57 | 0.997 | 0.003 | 1, 9.6 | 0.955 | 0.31 |
|  | Treatment | 2.293 | 1, 57 | 0.136 | 1.275 | 1, 11.7 | 0.281 |  |
|  | Sex | 2.447 | 1, 57 | 0.123 | 1.026 | 1, 9.6 | 0.336 |  |
|  | Diet × treatment | 4.605 | 1, 57 | **0.036** | 2.273 | 1, 11.7 | **0.158** |  |
|  | Diet × sex | 0.986 | 1, 57 | 0.325 | 0.388 | 1, 9.6 | 0.548 |  |
|  | Treatment × sex | 0.298 | 1, 57 | 0.587 | 0.126 | 1, 11.7 | 0.729 |  |
|  | Diet × treatment × sex | 1.429 | 1, 57 | 0.237 | 0.809 | 1, 11.7 | 0.387 |  |
| Center time, challenge 2 (n = 65) | Diet | 2.703 | 1, 57 | 0.106 | 1.355 | 1, 9.7 | 0.272 | 0.31 |
|  | Treatment | 15.459 | 1, 57 | < 0.001 | 8.643 | 1, 11.7 | 0.013 |  |
|  | Sex | 3.081 | 1, 57 | 0.085 | 1.354 | 1, 9.7 | 0.273 |  |
|  | Diet × treatment | 6.836 | 1, 57 | **0.011** | 3.201 | 1, 11.7 | **0.100** |  |
|  | Diet × sex | 0.002 | 1, 57 | 0.963 | 0.005 | 1, 9.7 | 0.946 |  |
|  | Treatment × sex | 0.901 | 1, 57 | 0.347 | 0.617 | 1, 11.7 | 0.448 |  |
|  | Diet × treatment × sex | 1.649 | 1, 57 | 0.204 | 1.055 | 1, 11.7 | 0.325 |  |
| Distance, novel open field (n = 63) | Diet | 1.577 | 1, 55 | 0.214 | 1.234 | 1, 9.6 | 0.294 | 0.07 |
|  | Treatment | 37.193 | 1, 55 | < 0.001 | 30.951 | 1, 12.1 | < 0.001 |  |
|  | Sex | 0.023 | 1, 55 | 0.880 | 0.019 | 1, 9.6 | 0.893 |  |
|  | Diet × treatment | 0.003 | 1, 55 | 0.957 | 0.003 | 1, 12.1 | 0.960 |  |
|  | Diet × sex | 0.000 | 1, 55 | 0.988 | 0.000 | 1, 9.6 | 0.992 |  |
|  | Treatment × sex | 1.562 | 1, 55 | 0.217 | 1.362 | 1, 12.1 | 0.266 |  |
|  | Diet × treatment × sex | 0.619 | 1, 55 | 0.435 | 0.516 | 1, 12.1 | 0.486 |  |
| Center time, novel open field (n = 63) † | Diet | 5.892 | 1, 55 | 0.019 | 5.892 | 1, 55 | 0.019 | 0.00 |
|  | Treatment | 32.232 | 1, 55 | < 0.001 | 32.232 | 1, 55 | < 0.001 |  |
|  | Sex | 6.635 | 1, 55 | 0.013 | 6.635 | 1, 55 | 0.013 |  |
|  | Diet × treatment | 0.212 | 1, 55 | 0.647 | 0.212 | 1, 55 | 0.647 |  |
|  | Diet × sex | 0.110 | 1, 55 | 0.741 | 0.110 | 1, 55 | 0.741 |  |
|  | Treatment × sex | 0.358 | 1, 55 | 0.552 | 0.358 | 1, 55 | 0.552 |  |
|  | Diet × treatment × sex | 2.004 | 1, 55 | 0.163 | 2.004 | 1, 55 | 0.163 |  |
| Center entries, novel open field (n = 63) | Diet | 3.968 | 1, 55 | 0.051 | 3.213 | 1, 10.0 | 0.103 | 0.07 |
|  | Treatment | 36.716 | 1, 55 | < 0.001 | 30.656 | 1, 12.6 | < 0.001 |  |
|  | Sex | 0.005 | 1, 55 | 0.943 | 0.005 | 1, 10.0 | 0.946 |  |
|  | Diet × treatment | 0.069 | 1, 55 | 0.794 | 0.058 | 1, 12.6 | 0.813 |  |
|  | Diet × sex | 0.011 | 1, 55 | 0.919 | 0.009 | 1, 10.0 | 0.924 |  |
|  | Treatment × sex | 1.311 | 1, 55 | 0.257 | 1.116 | 1, 12.6 | 0.311 |  |
|  | Diet × treatment × sex | 0.427 | 1, 55 | 0.516 | 0.359 | 1, 12.6 | 0.560 |  |

*† The between-cage variance was estimated at zero and the random intercept was therefore removed; the model reduces to the fixed-effects ANOVA.*

**Supplementary Table S2. Effect sizes for all ANOVA effects.**

Partial eta-squared (ηp²) for every effect of the three-way ANOVAs reported in the main text (diet × treatment × sex). For the treatment block and body weight, values are the between-subjects effects of the repeated-measures ANOVA (i.e., for the mean across sessions). Significant effects (p < 0.05) are shown in bold. Distance in cm; center time in s; center entries as counts; body weight in g or as a percentage of the pre-diet baseline.

| **Outcome** | **Effect** | **F** | **df** | **p** | **ηp²** |
| --- | --- | --- | --- | --- | --- |
| Distance, treatment block (between-subjects effects) | Diet | 6.719 | 1, 57 | **0.012** | 0.105 |
|  | Treatment | 10.077 | 1, 57 | **0.002** | 0.150 |
|  | Sex | 0.464 | 1, 57 | 0.498 | 0.008 |
|  | Diet × treatment | 1.071 | 1, 57 | 0.305 | 0.018 |
|  | Diet × sex | 0.077 | 1, 57 | 0.782 | 0.001 |
|  | Treatment × sex | 2.057 | 1, 57 | 0.157 | 0.035 |
|  | Diet × treatment × sex | 0.270 | 1, 57 | 0.605 | 0.005 |
| Center time, treatment block (between-subjects effects) | Diet | 4.419 | 1, 57 | **0.040** | 0.072 |
|  | Treatment | 11.680 | 1, 57 | **0.001** | 0.170 |
|  | Sex | 2.280 | 1, 57 | 0.137 | 0.038 |
|  | Diet × treatment | 6.043 | 1, 57 | **0.017** | 0.096 |
|  | Diet × sex | 1.132 | 1, 57 | 0.292 | 0.019 |
|  | Treatment × sex | 0.064 | 1, 57 | 0.802 | 0.001 |
|  | Diet × treatment × sex | 0.510 | 1, 57 | 0.478 | 0.009 |
| Distance, challenge 1 | Diet | 1.554 | 1, 57 | 0.218 | 0.027 |
|  | Treatment | 8.666 | 1, 57 | **0.005** | 0.132 |
|  | Sex | 0.742 | 1, 57 | 0.393 | 0.013 |
|  | Diet × treatment | 1.805 | 1, 57 | 0.184 | 0.031 |
|  | Diet × sex | 2.814 | 1, 57 | 0.099 | 0.047 |
|  | Treatment × sex | 2.659 | 1, 57 | 0.108 | 0.045 |
|  | Diet × treatment × sex | 4.790 | 1, 57 | **0.033** | 0.078 |
| Center time, challenge 1 | Diet | 1.114 | 1, 57 | 0.296 | 0.019 |
|  | Treatment | 17.019 | 1, 57 | **< 0.001** | 0.230 |
|  | Sex | 2.436 | 1, 57 | 0.124 | 0.041 |
|  | Diet × treatment | 5.498 | 1, 57 | **0.023** | 0.088 |
|  | Diet × sex | 6.966 | 1, 57 | **0.011** | 0.109 |
|  | Treatment × sex | 0.668 | 1, 57 | 0.417 | 0.012 |
|  | Diet × treatment × sex | 4.048 | 1, 57 | **0.049** | 0.066 |
| Distance, challenge 2 | Diet | 0.000 | 1, 57 | 0.997 | 0.000 |
|  | Treatment | 2.293 | 1, 57 | 0.136 | 0.039 |
|  | Sex | 2.447 | 1, 57 | 0.123 | 0.041 |
|  | Diet × treatment | 4.605 | 1, 57 | **0.036** | 0.075 |
|  | Diet × sex | 0.986 | 1, 57 | 0.325 | 0.017 |
|  | Treatment × sex | 0.298 | 1, 57 | 0.587 | 0.005 |
|  | Diet × treatment × sex | 1.429 | 1, 57 | 0.237 | 0.024 |
| Center time, challenge 2 | Diet | 2.703 | 1, 57 | 0.106 | 0.045 |
|  | Treatment | 15.459 | 1, 57 | **< 0.001** | 0.213 |
|  | Sex | 3.081 | 1, 57 | 0.085 | 0.051 |
|  | Diet × treatment | 6.836 | 1, 57 | **0.011** | 0.107 |
|  | Diet × sex | 0.002 | 1, 57 | 0.963 | 0.000 |
|  | Treatment × sex | 0.901 | 1, 57 | 0.347 | 0.016 |
|  | Diet × treatment × sex | 1.649 | 1, 57 | 0.204 | 0.028 |
| Distance, novel open field | Diet | 1.577 | 1, 55 | 0.214 | 0.028 |
|  | Treatment | 37.193 | 1, 55 | **< 0.001** | 0.403 |
|  | Sex | 0.023 | 1, 55 | 0.880 | 0.000 |
|  | Diet × treatment | 0.003 | 1, 55 | 0.957 | 0.000 |
|  | Diet × sex | 0.000 | 1, 55 | 0.988 | 0.000 |
|  | Treatment × sex | 1.562 | 1, 55 | 0.217 | 0.028 |
|  | Diet × treatment × sex | 0.619 | 1, 55 | 0.435 | 0.011 |
| Center time, novel open field | Diet | 5.892 | 1, 55 | **0.019** | 0.097 |
|  | Treatment | 32.232 | 1, 55 | **< 0.001** | 0.369 |
|  | Sex | 6.635 | 1, 55 | **0.013** | 0.108 |
|  | Diet × treatment | 0.212 | 1, 55 | 0.647 | 0.004 |
|  | Diet × sex | 0.110 | 1, 55 | 0.741 | 0.002 |
|  | Treatment × sex | 0.358 | 1, 55 | 0.552 | 0.006 |
|  | Diet × treatment × sex | 2.004 | 1, 55 | 0.163 | 0.035 |
| Center entries, novel open field | Diet | 3.968 | 1, 55 | 0.051 | 0.067 |
|  | Treatment | 36.716 | 1, 55 | **< 0.001** | 0.400 |
|  | Sex | 0.005 | 1, 55 | 0.943 | 0.000 |
|  | Diet × treatment | 0.069 | 1, 55 | 0.794 | 0.001 |
|  | Diet × sex | 0.011 | 1, 55 | 0.919 | 0.000 |
|  | Treatment × sex | 1.311 | 1, 55 | 0.257 | 0.023 |
|  | Diet × treatment × sex | 0.427 | 1, 55 | 0.516 | 0.008 |
| Body weight, days 1–57 (between-subjects effects) | Diet | 0.225 | 1, 57 | 0.637 | 0.004 |
|  | Treatment | 0.025 | 1, 57 | 0.874 | 0.000 |
|  | Sex | 405.797 | 1, 57 | **< 0.001** | 0.877 |
|  | Diet × treatment | 0.080 | 1, 57 | 0.779 | 0.001 |
|  | Diet × sex | 0.037 | 1, 57 | 0.847 | 0.001 |
|  | Treatment × sex | 0.007 | 1, 57 | 0.933 | 0.000 |
|  | Diet × treatment × sex | 0.110 | 1, 57 | 0.742 | 0.002 |
| Body weight, % of baseline (between-subjects effects) | Diet | 2.637 | 1, 57 | 0.110 | 0.044 |
|  | Treatment | 0.090 | 1, 57 | 0.766 | 0.002 |
|  | Sex | 4.647 | 1, 57 | **0.035** | 0.075 |
|  | Diet × treatment | 0.475 | 1, 57 | 0.493 | 0.008 |
|  | Diet × sex | 0.029 | 1, 57 | 0.866 | 0.001 |
|  | Treatment × sex | 0.047 | 1, 57 | 0.829 | 0.001 |
|  | Diet × treatment × sex | 2.057 | 1, 57 | 0.157 | 0.035 |

**Supplementary Table S3. Post hoc comparisons of diet within each treatment condition, separately for males and females.**

For each outcome with a significant diet × treatment or higher-order interaction in the main text, the simple main effect of diet (Kava − Control) was tested within the saline and within the nicotine condition, separately for males and females, using the error term of the two-way (diet × treatment) ANOVA within each sex. n is given as Kava, Control. p (uncorrected) is the simple-effect p-value; p (Bonferroni) and the 95% confidence interval are adjusted for the two comparisons (saline and nicotine) made within each sex. Significant comparisons after correction are shown in bold. Distance in cm; center time in s (treatment block values are the mean of the five sessions).

| **Outcome** | **Sex** | **Treatment** | **n** | **Mean difference (Kava − Control)** | **SE** | **p (uncorrected)** | **p (Bonferroni)** | **95% CI (Bonferroni)** |
| --- | --- | --- | --- | --- | --- | --- | --- | --- |
| Center time, treatment block | Males | Saline | 8, 8 | **140.0** | 46.7 | 0.006 | **0.011** | 29.6, 250.4 |
|  | Males | Nicotine | 8, 9 | -0.1 | 45.4 | 0.998 | 1.000 | -107.5, 107.2 |
|  | Females | Saline | 8, 8 | 61.4 | 42.0 | 0.155 | 0.310 | -38.1, 161.0 |
|  | Females | Nicotine | 8, 8 | -15.6 | 42.0 | 0.714 | 1.000 | -115.2, 84.0 |
| Distance, challenge 1 | Males | Saline | 8, 8 | -265.8 | 397.2 | 0.509 | 1.000 | -1204.6, 673.1 |
|  | Males | Nicotine | 8, 9 | 86.4 | 386.0 | 0.825 | 1.000 | -826.0, 998.8 |
|  | Females | Saline | 8, 8 | **1344.9** | 441.5 | 0.005 | **0.010** | 299.2, 2390.6 |
|  | Females | Nicotine | 8, 8 | -126.6 | 441.5 | 0.776 | 1.000 | -1172.3, 919.1 |
| Center time, challenge 1 | Males | Saline | 8, 8 | -35.8 | 58.7 | 0.547 | 1.000 | -174.5, 102.9 |
|  | Males | Nicotine | 8, 9 | -54.8 | 57.0 | 0.344 | 0.688 | -189.7, 80.0 |
|  | Females | Saline | 8, 8 | **230.4** | 56.5 | < 0.001 | **< 0.001** | 96.5, 364.3 |
|  | Females | Nicotine | 8, 8 | -18.9 | 56.5 | 0.740 | 1.000 | -152.8, 115.0 |
| Distance, challenge 2 | Males | Saline | 8, 8 | 439.1 | 396.5 | 0.277 | 0.554 | -498.1, 1376.3 |
|  | Males | Nicotine | 8, 9 | 8.7 | 385.3 | 0.982 | 1.000 | -902.1, 919.5 |
|  | Females | Saline | 8, 8 | 530.8 | 510.2 | 0.307 | 0.614 | -677.7, 1739.2 |
|  | Females | Nicotine | 8, 8 | -981.9 | 510.2 | 0.065 | 0.129 | -2190.3, 226.6 |
| Center time, challenge 2 | Males | Saline | 8, 8 | 89.8 | 62.2 | 0.160 | 0.319 | -57.2, 236.9 |
|  | Males | Nicotine | 8, 9 | 10.7 | 60.5 | 0.861 | 1.000 | -132.2, 153.6 |
|  | Females | Saline | 8, 8 | **163.5** | 57.4 | 0.008 | **0.016** | 27.5, 299.4 |
|  | Females | Nicotine | 8, 8 | -68.4 | 57.4 | 0.243 | 0.486 | -204.4, 67.5 |
